# Effect of dapagliflozin on readmission and loop diuretics use in patients with acute heart failure: a retrospective propensity score-matched cohort study

**DOI:** 10.1186/s12872-024-04078-5

**Published:** 2024-08-02

**Authors:** Dong Wu, Zhen Ma, Xiaoying Wang, Xiaowu Wang, Xiaojuan Wang

**Affiliations:** 1 Department of Pharmacy, Fuyang People’s Hospital, Fuyang, Anhui China; 2 Department of Cardio Vascular Medicine, Fuyang People’s Hospital, Fuyang, Anhui China; 3 Department of Clinical Laboratory, The Second People’s Hospital of Fuyang, Fuyang infection Disease Clinical College of Anhui Medical university, Fuyang, Anhui China

**Keywords:** Dapagliflozin, Loop diuretics, Readmission, Acute heart failure, Renal function

## Abstract

**Background:**

The efficacy of dapagliflozin in patients with acute heart failure remains unclear.

**Objective:**

To investigate the impact of dapagliflozin (DAPA) on loop diuretics use and 90-day readmission in patients with acute heart failure.

**Methods:**

In a retrospective cohort study, patients diagnosed with acute heart failure or chronic heart failure with acute exacerbation admitted to Fuyang People’s Hospital from January 2021 to April 2023, this study used DAPA (at a dose of 10 mg once daily) in combination with standard treatment. The patients were divided into DAPA group and DAPA-Free group based on whether they used DAPA in acute heart failure. To minimize the influence of confounding factors and ensure comparability between groups, we used propensity score matching (PSM).

**Results:**

A total of 399 patients were included, with 206 patients (51.63%) in the DAPA group and 193 patients (48.37%) in the DAPA-Free group. PSM produced 160 pairs. After PSM, there were no statistically significant differences between the DAPA and DAPA-Free groups in terms of readmission of all causes (16.88% vs. 18.12%, OR 0.9141, 95% CI 0.5385–1.552, log rank *P* = 0.739) or readmission for heart failure (11.88% vs. 15.0%, OR 0.9077, 95% CI 0.4441–1.469, log rank *P* = 0.484) after 90-day follow-up. Patients in the DAPA group had a lower mean daily dose of intravenous loop diuretics compared to the DAPA-Free group (20 mg/d vs. 30.00 mg/d, *P*<0.001), lower total loop diuretic dose during hospitalization (106.06 ± 31.23 mg vs. 144.50 ± 45.39 mg, *P* = 0.038) and a decreased number of diuretic types used (11.88% vs. 23.12%, *P* = 0.008).

**Conclusions:**

DAPA reduced the dose of intravenous loop diuretics. However, it did not improve all-cause readmission for 90 days or readmission for heart failure after discharge.

## Introduction

Acute heart failure is a life-threatening condition characterized by a sudden onset of symptoms due to the inability of the heart to pump sufficient blood to meet the body’s metabolic demands [1]. In most cases of acute heart failure, patients show signs of fluid overload upon admission and are commonly managed with a progressive increase in intravenous loop diuretic therapy to alleviate symptoms and minimize complications [2]. Unfortunately, this treatment approach is often hampered by the deterioration of renal function and does not achieve sufficient decongestion for many patients during their hospitalization for acute heart failure [3, 4]. Thus, there is a prevalent and complex clinical syndrome associated with high morbidity and mortality rates. Within 30 days of discharge, Au AG et al. reported that 19% of patients with heart failure had an unplanned readmission [5], and 44% of patients readmitted to the hospital at least once in 6 months [6]. Based on the study conducted by Kimmoun A, all-cause mortality rates at 30 days and 1 year were reported to be 7% and 24%, respectively [7]. Patients with acute heart failure experience high rates of readmission and hospital mortality, imposing a significant burden on society and families [8]. Unfortunately, current treatment for acute heart failure has not changed significantly in decades. Therefore, there is a clinical need for new therapeutic strategies in this population.

Over the years, various treatment strategies have been used to address the management of acute heart failure. Sodium–glucose cotransporter 2 (SGLT-2) inhibitors have emerged as a promising class of drugs for the management of chronic heart failure and have become a cornerstone of guideline-recommended pharmacological therapy [2, 9]. SGLT-2 inhibitors have gained considerable attention and are increasingly being used in China as an effective treatment option for diabetes and, to some extent, for the management of chronic kidney disease (CKD). Numerous studies have shown their efficacy in achieving glycemic control, reducing cardiovascular risks, and providing renoprotective benefits. In the context of diabetes management, SGLT2 inhibitors have shown positive results by lowering hemoglobin A1c levels, promoting weight loss, and improving blood pressure control in Chinese patients [10–12]. These medications have also been associated with possible renoprotective effects, including attenuation of albuminuria and delayed reduction in renal function in patients with diabetes and CKD [13]. Therefore, SGLT2 inhibitors are increasingly being used in patients with chronic heart failure combined with type 2 diabetes or CKD. DAPA, a selective SGLT2 inhibitor, originally approved for the treatment of type 2 diabetes mellitus, has shown favorable effects on cardiovascular outcomes, especially in patients with chronic heart failure and reduced ejection fraction [14]. The mechanism of action of DAPA involves the selective inhibition of SGLT2, which results in increased urinary glucose excretion and subsequent reduction in plasma glucose levels. However, beyond its glucose-lowering effects, DAPA has demonstrated additional benefits in heart failure, including increased urine excretion, improved functional capacity, and a possible protective effect on renal function [15]. Numerous clinical trials, including landmark trials such as DAPA-HF [16] and DECLARE-TIMI 58 Trial [17], have investigated the efficacy and safety of DAPA in patients with heart failure. These studies have consistently shown a reduction in the risk of chronic heart failure hospitalizations and cardiovascular death, as well as improvements in symptoms and quality of life.

SGLT2 inhibitors have shown potential as a treatment option for acute heart failure based on their underlying mechanisms. However, there is limited and conflicting evidence on their clinical usefulness. For example, the EMPA-RESPONSE-AHF study [18] conducted in patients with acute heart failure found that empagliflozin treatment did not have a significant impact on the change in the severity of dyspnea, the diuretic response, the levels of NT-proBNP, or the length of hospital stay. In contrast, Ibrahim et al. reported that DAPA had a significant diuretic effect in diabetic patients [19]. None of the studies included in the analysis administered SGLT-2 inhibitors in an early stage of acute heart failure treatment.

Taking into account the possible diuretic and natriuretic effects of DAPA, it is reasonable to hypothesize that early initiation of DAPA can lead to better symptomatic improvement in patients with AHF. Thus, this study aimed to primarily assess the efficacy and safety of DAPA on loop diuretics use and 90-readmission in patients with acute heart failure.

## Methods

### Study design

A single center retrospective cohort study was conducted at Fuyang People’s Hospital to compare propensity scores.

### Study population

The study involved patients over 18 years of age, diagnosed with acute heart failure or acute worsening of chronic heart failure, and receiving intravenous loop diuretic therapy. The diagnosis was based on the Heart Failure Guidelines of the European Society of Cardiology [20]. Patients were divided into two groups based on whether they received DAPA during the acute stage (DAPA was started within 6 days after admission): the DAPA group and the DAPA-Free group. The permission to conduct the study was approved by the Ethics Committee of Fuyang People’s Hospital [NO.202151].

Exclusion criteria were as follows: (1) non-use of intravenous loop diuretic drugs; (2) acute heart failure combined with acute myocardial infarction; (3) prior use of SGLT2i, discontinue use for less than 4 weeks; (4) Ccr < 30mL/min; (5) cardiogenic shock; (6) mechanical ventilation; (7) Child-Pugh class C liver failure; (8) DAPA group patients who discontinued DAPA use within 90 days after hospital discharge. (9) absence of relevant data such as creatinine; (10) death during hospitalization.

### Research protocol

In addition to standard therapy, patients in the DAPA group received a daily dose of DAPA at 10 mg. Meanwhile, patients in the DAPA-Free group received standard therapy for acute heart failure.

The clinical data collected includes demographic information (such as age, sex and length of hospital stay), underlying diseases (such as coronary heart disease, history of percutaneous coronary intervention [PCI], and chronic obstructive pulmonary disease [COPD]), laboratory parameters (including hemoglobin, serum creatinine, and NT-proBNP), concomitant medications (such as angiotensin receptor blocker [ARB], calcium channel blocker [CCB], and oral anticoagulants), intravenous loop diuretic use (encompassing diuretic type, dosage, potential dosage increments, and the number of diuretics), and safety events (including symptomatic hypotension, hypoglycemia, ketoacidosis and acute kidney injury).

The total diuretic dose of the loop was defined as the equivalent dose of furosemide, with conversion standards established as follows: 1 mg bumetanide = 20 mg torasemide = 40 mg furosemide. The diuretic loop daily dose was defined as the total diuretic dose divided by the number of days the diuretics were administered. Oral anticoagulant medication is defined as the use of any of the following: warfarin, dabigatran, rivaroxaban, or apixaban. Acute kidney injury was defined according to the KDIGO criteria as an increase in serum creatinine greater than 26.50 µmol/L (0.3 mg / dl) in 48 h. DAPA-related safety events were defined as symptomatic hypotension, hypoglycemia, oliguria, ketosis, and acute kidney injury.

### Study endpoints

The primary endpoint was all-cause readmission and readmission for heart failure. Secondary endpoints were total loop diuretics doses and renal function deterioration during hospitalization.

### Follow-up after discharge

The follow-up data were provided by the Heart Failure Center of Fuyang People’s Hospital. A follow-up visit was made 30 days ± 7 days, 60 days ± 7 days, and 90 days ± 7 days after hospital discharge. The follow-up inquiry focused on whether the patient had been readmitted and the cause of his readmission.

### Sample size estimation

The sample size was estimated using the PASS 11 software. Group sample sizes of 185 in the DAPA group and 185 in the DAPA-Free group achieve 90% power to detect a difference between the group proportions of 0.15. The proportion in the DAPA group is assumed to be 0.35 under the null hypothesis and 0.20 under the alternative hypothesis. The proportion in the DAPA-Free group is 0.35. The test statistic used is the Fisher exact two-sided test. The significance level of the test was 0.05.

### Statistical analysis

SPSS 26.0 was utilized for data analysis in this study. For normally distributed data, a t-test was used to compare the means between groups. Continuous data were represented as mean ± standard deviation. When data deviated from the normal distribution, a Mann-Whitney U test was used for intergroup comparisons and the median (P25, P75) was used to represent ordinal data. Categorical data were analyzed using either a chi-square test or Fisher’s exact test. GraphPad Prism 5 was used to generate follow-up curves for the two groups of patients. Propensity score matching (PSM) was used to minimize the bias of confounding factors and facilitate comparability between groups. Atrial fibrillation, heart valve disease, diastolic blood pressure (DBP), hemoglobin, LVEF, fasting blood glucose, and Killip heart functional classification were included as matching variables. A 1:1 PSM approach with greedy nearest-neighbor matching and a caliper of 0.02 was utilized for sex matching to eliminate bias and account for potential confounders’ impact. A significance level of *P* < 0.05 was considered statistically significant.

## Results

### Baseline characteristics

**Fig. 1:**
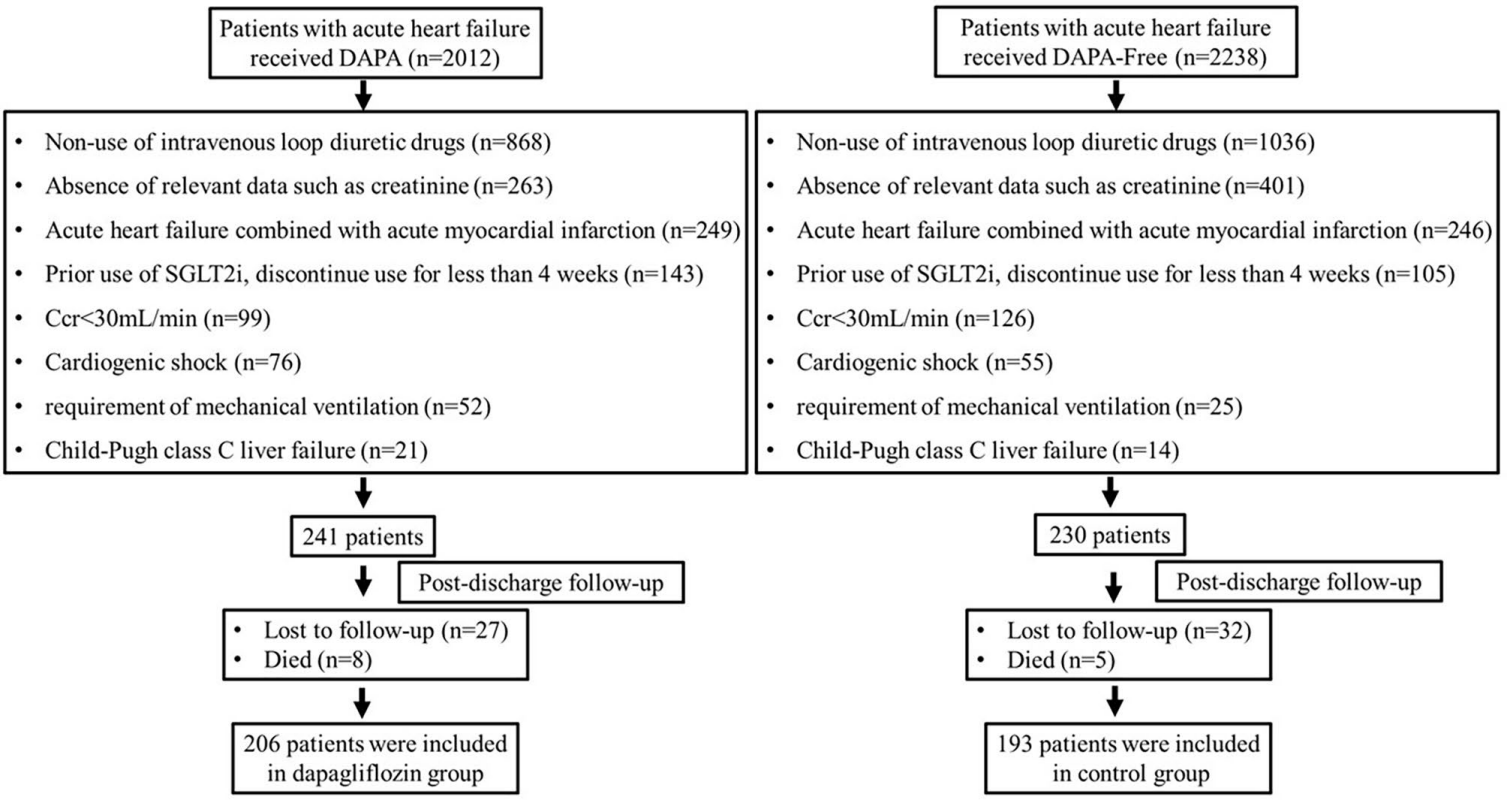
Flow chart of patient screening and selection process. (Ccr: creatinine clearance rate; SGLT2i: sodium-glucose cotransporter-2 inhibitor)

From January 2021 to April 2023, a total of 2012 patients in the DAPA group and 2238 patients in the DAPA-Free group with a clinical diagnosis of acute heart failure and who met the inclusion / exclusion criteria were hospitalized at Fuyang People’s Hospital. However, a total of 399 eligible patients were included, 206 patients (51.67%) were included in the DAPA group, while 193 patients (48.37%) were in the DAPA-Free group. The main exclusion criteria were non-use of intravenous loop diuretics, incomplete data, and acute myocardial infarction combined with acute heart failure, see Fig. 1.

Table 1 shows the baseline characteristics of the two groups. The median duration of dapagliflozin use for the patients was 2.00 (1.00, 3.00) days after admission. Before PSM, there were no significant differences in sex, age, hospital stay, comorbidity index, coronary heart disease, PCI, hypertension, COPD, anemia, SBP, HR, CCr at admission or some oral drugs. The DAPA group had significantly fewer patients with atrial fibrillation [36.41% vs. 48.19%, *P* = 0.017] and heart valve disease [15.05% vs. 23.83%, *P* = 0.026] than the DAPA-Free group. The severity of heart failure evaluated by the NYHA class and the level of LVEF was significantly different between the two groups. There were 105 patients (50.97%) with LVEF < 40% in the DAPA group and 88 patients (45.59%) in the DAPA-Free group. The proportion of LVEF < 40% patients was 48.37% before PSM. Regarding the laboratory findings, the hemoglobin and fasting blood glucose levels at admission were significantly higher in the DAPA group than in the DAPA-Free group. Additionally, patients in the DAPA group had higher diastolic blood pressure upon admission.

Using a 1:1 PSM, 160 patients taking DAPA were eventually matched with 160 patients who did not take DAPA. After PSM, the statistical tests demonstrated a successful match in all baseline patient characteristics and clinical variables (Table 1).

**Table 1:** Characteristics of the patients at baseline

Variable	Before PSM			After PSM		
	DAPA group(*n* = 206)	DAPA-Free group(*n* = 193)	*P* value	DAPA group(*n* = 160)	DAPA-Free group(*n* = 160)	*P* value
Sex (male)	102(49.51)	96(49.74)	0.964	75(46.88)	80(50.00)	0.576
Age (years)	75.00(60.75, 79.25)	75.00(67.00,82.00)	0.121	77.00(65.25, 80.00)	74.00(63.50, 81.00)	0.545
Hospital stay	8.00(7.00, 10.00)	8.00(7.00,10.00)	0.618	9.00(7.00, 11.00)	8.00(7.00,11.00)	0.600
Comorbidity index	3.00(2.00, 4.00)	3.00(2.00,4.00)	0.908	3.00(2.00, 4.00)	3.00(2.00, 4.00)	0.482
Coronary heart disease	161(78.16)	145(75.13)	0.475	120(75.00)	110(68.75)	0.214
PCI	39(18.93)	27(13.99)	0.184	29(18.12)	19(11.88)	0.117
Atrial fibrillation	75(36.41)	93(48.19)	0.017	72(45.00)	74(46.25)	0.822
Hypertension	115(55.82)	120(62.18)	0.198	88(55.00)	99(61.87)	0.212
Type 2 diabetes	102(49.51)	89(46.11)	0.497	81(50.62)	75(46.88)	0.502
COPD	22(10.68)	23(11.92)	0.696	21(13.12)	17(10.62)	0.489
Heart valve disease	31(15.05)	46(23.83)	0.026	27(16.88)	33(20.62)	0.390
Anemia	31(15.05)	27(13.99)	0.764	29(18.12)	19(11.88)	0.117
NYHA class			0.003			0.212
Class II	26(12.62)	13(6.74)		22(13.75)	13(8.12)	
Class III	132(64.08)	153(79.27)		111(69.38)	123(76.88)	
Class IV	48(23.30)	27(13.99)		27(16.88)	24(15.00)	
SBP at, mm Hg	131.50(113.75, 148.00)	128.00(112.00,151.00)	0.422	130.00(114.00, 147.00)	131.50(113.00, 153.00)	0.699
DBP at, mm Hg	77.50(64.75, 89.00)	72.00(60.00,83.00)	0.004	73.50(64.00, 86.00)	74.00(62.00, 86.00)	0.829
HR (beats/min)	83.50(74.00, 99.00)	83.00(69.00,98.00)	0.240	81.00(72.00, 98.00)	84.50(70.00, 98.00)	0.954
NT-proBNP, pg/mL	2941.00(1389.00, 8045.00)	2392.00(6438.00,965.00)	0.243	2453.00(1142.25, 7833.25)	2629.50(1056.75, 6471.00)	0.934
NT-proBNP at discharge, pg/mL	843.00(146.00, 1324.00)	905.00(183.00, 1434.00)	0.630	795.00(164.00, 1280.00)	840.00(175.00, 1324.00)	0.742
Hemoglobin, g/L	111.00(70.75, 130.00)	122.00(108.50,138.00)	0.001	109.00(70.00, 130.00)	113.00(97.00,133.00)	0.402
CCr on admission, mL/min	53.65(39.22, 71.70)	55.54(37.27,74.29)	0.900	52.30(36.65, 69.05)	56.70(40.88, 78.20)	0.127
Fasting blood glucose, mmol/L	6.03(4.82, 8.42)	5.54(4.46,6.47)	0.001	6.11(5.07, 8.53)	5.92(4.36, 6.45)	0.225
LVEF, %	40.00(29.00, 52.25)	48.00(37.25,57.00)	0.001	43.00(33.00, 55.00)	43.00(39.00, 52.00)	0.368
≥40%	101(49.03)	105(54.40)	0.283	83(51.88)	86(53.75)	0.737
<40%	105(50.97)	88(45.59)		77(48.12)	74(46.25)	
Loop diuretics	206(100.00)	193(100.00)	/	160(100.00)	160(100.00)	/
Beta-blocker	125(60.68)	106(54.92)	0.244	92(57.50)	87(54.38)	0.573
CCB	31(15.05)	27(13.99)	0.764	22(13.75)	23(14.38)	0.872
ARNI/ACEI/ARB	160(77.67)	137(70.98)	0.126	120(75.00)	117(73.12)	0.702
Antiarrhythmic drugs	33(16.02)	21(10.88)	0.114	27(16.88)	18(11.25)	0.148
Oral anticoagulant	60(29.13)	71(36.79)	0.134	54(33.75)	56(35.00)	0.814
Digoxin	62(30.09)	58(30.05)	0.992	55(34.38)	48(30.00)	0.402
Cholesterol inhibitor	180(87.38)	163(84.46)	0.401	142(88.75)	133(83.12)	0.148
Mineralocorticoid receptor antagonist	123(59.71)	106(54.92)	0.334	102(63.75)	97(60.62)	0.564

### Postdischarge follow-up

**Fig. 2:**
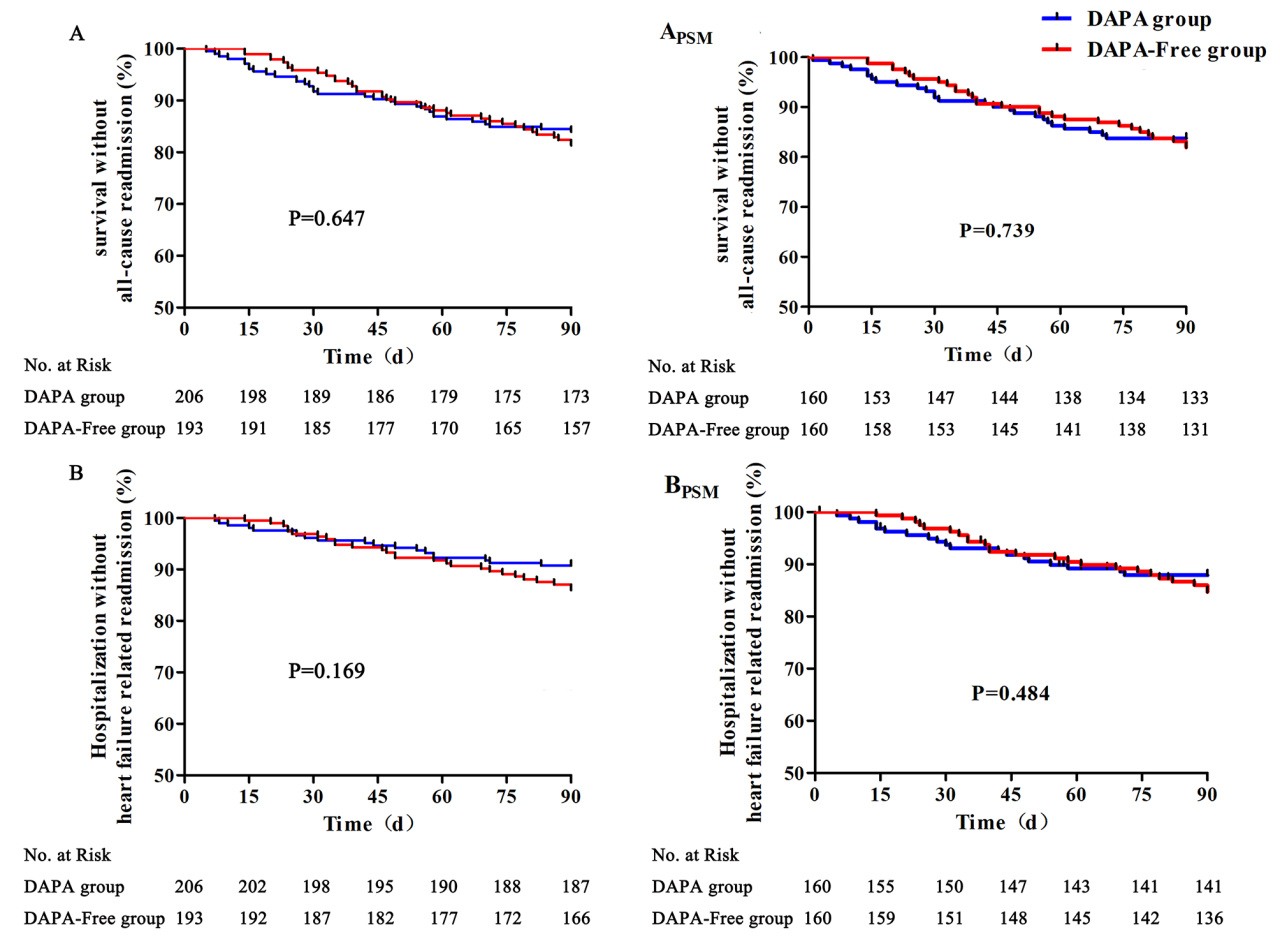
Survival analysis for readmission in two group patients. (**A**: all-cause readmission of before PSM; **B**: heart failure related readmission of before PSM; A_PSM_ all-cause readmission of after PSM; B_PSM_: heart failure related readmission of after PSM)

All 399 patients were followed up for 90 days, and a total of 69 patients were re-hospitalized, including 33 cases in the DAPA group and 36 cases in the DAPA-Free group. After the 90-day follow-up period, a total of 11 patient deaths were observed, 6 in the DAPA group and 5 in the DAPA-Free group. After PSM, a total of 27 cases of readmission were recorded in the DAPA group, while the DAPA-Free group had 29 readmission cases. All-cause readmission rates between the two groups did not show statistically significant differences (16.88% vs. 18.12%, OR 0.9141, 95% CI 0.5385–1.552, log-rank *P* = 0.739). In terms of readmission due to heart failure, 19 cases were observed in the DAPA group, compared to 24 cases in the DAPA-Free group. The difference in readmission rates for heart failure between the two groups was not statistically significant (11.88% vs. 15.00%, OR 0.9077, 95% CI 0.4441–1.469, log rank *P* = 0.484) (Fig. 2).

### Outcomes of hospitalization

The outcomes of hospitalization before and after PSM were summarized in Table 2. After PSM, patients in the DAPA group had a lower median daily dose (20 mg/d vs. 30.00 mg/d, *P*<0.001), reduced total loop diuretic doses during hospitalization (106.06 ± 31.23 mg vs. 144.50 ± 45.39 mg, *P* = 0.038), and a lower percentage of patients who required an increase in the number of diuretic types (11.88% vs. 23.12%, *P* = 0.008). However, there were no statistically significant differences between the two groups in terms of the need for an increase in diuretic dosage (20.62% vs. 23.75%, *P* = 0.501) and duration of loop diuretic (median: 2.00 days vs. 2.00 days, *P* = 0.959 )(Table 2).

**Table 2:** Effects of loop diuretics in two groups of patients

Variable	Before PSM			After PSM		
	DAPA group(*n* = 206)	DAPA-Free group (*n* = 193)	*P* value	DAPA group (*n* = 160)	DAPA-Free group (*n* = 160)	*P* value
Loop diuretic daily dose, mg/d	20.00(20.00, 31.57)	30.00(20.00, 40.00)	0.001	20.00(20.00, 30.00)	30.00(20.00, 40.00)	<0.001
Total doses of loop diuretics during hospitalisation (in furosemide equivalents), mg	114.85 ± 33.84	140.71 ± 41.69	0.041	106.06 ± 31.23	144.50 ± 45.39	0.038
Increasing the dose of loop diuretics, n (%)	40(19.42)	42(21.76)	0.563	33(20.62)	38(23.75)	0.501
Duration of loop diuretic, d	2.00(1.00, 5.00)	2.00(1.00, 4.50)	0.369	2.00(1.00, 4.75)	2.00(1.00, 5.00)	0.959
Adding another class of diuretic drugs, n (%)	24(11.65)	41(21.24)	0.010	19(11.88)	37(23.12)	0.008

### Safety

Information about safety events was summarized in Table 3. After PSM, the incidence of safety events was mainly deterioration of renal function and symptomatic hypotension, but there were no statistically significant differences in the rates of deterioration of renal function (DAPA group: 5.00%; DAPA-Free group:4.38%, *P* = 0.791) and symptomatic hypotension (DAPA group: 3.75%; DAPA-Free group: 5.62%, *P* = 0.428) between the two groups. Furthermore, no significant reduction in Ccr was observed in the DAPA group during the period under observation (from admission to discharge). The change in Ccr was 1.94 (− 7.39; 16.88) ml / min in the DAPA-Free group compared to 2.52(− 4.59; 11.15) ml / min in the DAPA-Free group (*P* = 0.770). Both groups had low rates of hypoglycemia, ketoacidosis, and urinary tract infections, and there were no statistically significant differences were observed (*P* > 0.05). There were no statistically significant differences between the two groups of patients in terms of overall safety events (DAPA group: 12.50%; DAPA-Free group: 13.75%, *P* = 0.741).

**Table 3:** Safety events

Event	Before PSM			After PSM		
	DAPA group (*n* = 206)	DAPA-Free group (*n* = 193)	*P* value	DAPA group (*n* = 160)	DAPA-Free group (*n* = 160)	*P* value
Deterioration of renal function, n (%)	19(9.22)	11(5.70)	0.182	8(5.00)	7(4.38)	0.791
Symptomatic hypotension, n (%)	8(3.88)	10(5.18)	0.532	6(3.75)	9(5.62)	0.428
Hypoglycemia	2(0.97)	5(2.59)	0.218	2(1.25)	4(2.50)	0.680
Ketoacidosis, n (%)	2(0.97)	1(0.52)	1.000	2(1.25)	1(6.25)	0.558
Urinary tract infections, n (%)	2(0.97)	1(0.52)	1.000	2(1.25)	1(6.25)	0.558
total	33(16.02)	28(14.51)	0.675	20(12.50)	22(13.75)	0.741

## Discussion

The use of diuretics in the treatment of acute heart failure is a fundamental component of therapy to alleviate symptoms of fluid overload. However, excessive reliance on diuretics in acute heart failure patients poses several challenges, including diuretic resistance, electrolyte imbalances, renal dysfunction, and detrimental effects on long-term outcomes [21, 22]. As such, there is a need to explore alternative treatment strategies that can reduce diuretic use while maintaining or improving clinical efficacy. DAPA exerts its primary effect by reducing renal re-absorption of glucose and sodium, leading to increased glycosuria and natriuresis [23]. This unique mechanism of action suggests that DAPA may have the potential to reduce the need for diuretics in patients with AHF.

Our study demonstrates that, compared to the DAPA-Free group, patients in the DAPA group showed an improved response to loop diuretics, manifested primarily as a lower daily dose (*P* < 0.001), a reduced total diuretic dose during hospitalization (*P* = 0.038), and a lower proportion of patients requiring increased diuretic types (*P* = 0.008). In a pilot study, a notable distinction was observed in the average daily doses of loop diuretics administered during hospital stay, the DAPA group exhibiting significantly lower dosages (78.46 ± 38.95 mg/day) compared to the DAPA-Free group (102.82 ± 31.26 mg/day, *P* = 0.001). Additionally, the DAPA group demonstrated a reduced proportion of patients requiring escalation to higher daily doses of loop diuretics (14% vs. 30%, *P* = 0.048) [24]. The EMPA-RESPONSE-AHF study has reported a decrease in the need to increase diuretic therapy with the use of SGLT2 inhibitors in patients with acute heart failure [18]. Furthermore, a retrospective analysis showed that early initiation of SGLT2 inhibitors in AHF and type 2 diabetes mellitus was associated with reduced doses of loop diuretics [25]. These studies consistently demonstrated a reduction in diuretic requirements and loop diuretic doses with DAPA therapy, suggesting that DAPA could act as a diuretic-sparing agent.

The observed diuretic-sparing effect of DAPA may have several clinical benefits. Firstly, it could help mitigate diuretic resistance, a common challenge encountered in the treatment of AHF [26]. By reducing the dependence on diuretics, DAPA can overcome the limitations posed by diuretic resistance and improve fluid management in these patients. Second, the reduction in diuretic use with DAPA may translate into a lower risk of electrolyte imbalances, specifically hypokalemia [27]. The preservation of potassium levels is particularly vital for patients with ischemic heart disease and those taking medications associated with QT prolongation [28, 29]. By minimizing the need for diuretics, DAPA could potentially reduce the occurrence of electrolyte disturbances and associated adverse events.

According to the reported that approximately one third of the patients could not achieve decongestion due to the development of acute cardiorenal syndrome, representing an acute deterioration of cardiac function, manifested by a decrease in kidney function [30]. In this study, the use of DAPA in patients with acute heart failure was not associated with a significant deterioration of renal function. The change in Ccr was 1.94 (− 7.39; 16.88) ml / min in the DAPA-Free group compared to 2.52 (− 4.59; 11.15) ml / min in the DAPA-Free group (*P* = 0.770). A meta-analysis of randomized trials free of DAPA found that there were no differences in the occurrence of worsening renal function between patients treated with or without SGLT2 inhibitors (OR 0.75; 95% CI 0.43–1.29; *P* = 0.290) [4]. However, Charaya K’s research [1] showed that the decrease in eGFR during hospitalization was more pronounced in the DAPA group (*P* = 0.049). They thought that a decrease in eGFR could be a reflection of successful decongestion [31]. Furthermore, some studies have shown that DAPA could improve renal function and reduce the biomarkers of renal injury in heart failure patients [32–34]. This preservation of renal function could be attributed to the effects of DAPA inhibition on the tubuloglomerular feedback mechanism, which reduces glomerular hyperfiltration and intrarenal congestion.

Existing research provided contradictory evidence regarding the rates of readmission due to heart failure at 30, 60, or 90 days after discharge for patients with acute heart failure who received DAPA treatment compared to placebo. The DAPA-RESPONSE-AHF study observed a significant decrease in hospital readmission rates within 30 days after discharge in the DAPA group compared to the placebo group. However, the sustainability of this reduction was not observed beyond day 60, as the intervention was implemented for a limited duration of only 30 days [35]. A prospective cohort study indicated that eligible patients for DAPA showed improved clinical outcomes in terms of all-cause mortality and rehospitalization compared to ineligible patients. The risk ratio (HR) for all-cause mortality or readmission in DAPA-eligible patients with acute decompensated heart failure remained lower compared to non-eligible patients, with an HR of 0.82 (95% CI 0.68–0.999, *P* = 0.049) [36].

In this study, DAPA treatment did not demonstrate a statistically significant difference in overall readmission rates or readmission rates due to heart failure compared to the DAPA-Free group in the 90-day follow-up study (*P* = 0.739 and *P* = 0.484, respectively). Our results were partially consistent with the SOLOIST-WHF study [37], which found a decrease in the primary endpoint, but did not include deaths resulting from cardiovascular causes. Although there was no statistically significant difference in overall readmission rates or readmission rates due to heart failure between the two groups of patients during the 90-day follow-up, the change trends observed in Fig. 2 indicate that, as time progressed, the readmission rate in the DAPA group was lower compared to the DAPA-Free group. This suggests that DAPA has the potential to reduce readmission rates in patients with chronic heart failure. More research is needed to evaluate the long-term effects after discharge.

This study has several limitations. First, due to its retrospective study, despite conducting multivariate analysis, there may still be residual confounding factors. Second, incomplete collection loss of follow-up after discharge may limit the interpretability of results for clinical endpoints. Third, the sample size is limited and the study cohort is derived from a single center, further restricting the generalizability of our findings. Lastly, the study included a small number of patients with LVEF < 40%, resulting in fewer patients taking medications such as β-blockers that improve prognosis. This could potentially affect the study outcomes. More prospective studies are warranted to validate these findings and explore the underlying mechanisms of DAPA’s impact on these outcomes.

## Conclusions

DAPA reduced the dose of intravenous loop diuretics without significantly increasing the incidence of deterioration of renal function deterioration. However, it did not improve all-cause readmission for 90 days or readmission for heart failure after discharge.

## Data Availability

Data Availability The data that support the findings of this study are available from the corresponding author upon reasonable request.

## Abbreviations

DAPA: Dapagliflozin
PSM: Propensity score matching
SGLT-2: Sodium–glucose cotransporter 2
CKD: Chronic kidney disease
PCI: Percutaneous coronary intervention
COPD: Chronic obstructive pulmonary disease
ARB: Angiotensin receptor blocker
CCB: Calcium channel blocker
